# Clinical and radiographic characteristics of presumptive tuberculosis patients previously treated for tuberculosis in Zambia

**DOI:** 10.1371/journal.pone.0263116

**Published:** 2022-01-27

**Authors:** Kondwelani Mateyo, Andrew D. Kerkhoff, Ian Dunn, Mutinta S. Nteeni, Monde Muyoyeta

**Affiliations:** 1 Department of Internal Medicine, University Teaching Hospital, Lusaka, Zambia; 2 Division of HIV, Infectious Diseases and Global Medicine, University of California San Francisco, San Francisco, California, United States of America; 3 Department of Radiology, University of British Columbia, Vancouver, Canada; 4 Department of Radiology, Levy Mwanawasa University Teaching Hospital, Lusaka, Zambia; 5 Centre for Infectious Disease Research in Zambia, Lusaka, Zambia; Rutgers Biomedical and Health Sciences, UNITED STATES

## Abstract

**Background:**

Persistent respiratory symptoms and radiographic abnormalities are common among individuals previously treated for tuberculosis (TB) and may contribute to misdiagnosis and incorrect treatment when they seek care. We sought to determine if clinical and radiographic characteristics differed among previously treated, presumptive TB patients according to their current TB disease status.

**Methods:**

Adults (>18 years of age) seeking care at a public health facility in Lusaka, Zambia were systematically evaluated for active TB using symptom screening and chest X-ray. All patients with presumptive TB submitted a sputum sample for microbiological TB testing. Patients who reported a prior history of TB treatment were included in the present analysis. ‘Confirmed TB’ was defined by the detection of TB using Xpert Ultra and/or liquid culture, while ‘possible TB’ was defined by the receipt of TB treatment without a positive Xpert Ultra or culture result. We evaluated the positive predictive value (PPV) of clinical symptoms and radiographic features for active TB alone and in combination.

**Results:**

Of 740 presumptive TB patients, 144 (19%) had been previously treated for active TB. Of these, 19 (13%) patients had confirmed TB, 14 (10%) had possible TB, and 111 (77%) had no pulmonary TB. Overall, 119 (83%) patients had ≥1 current respiratory symptom—this did not differ according to current TB disease classification (95%, 93%, 79%; p = 0.23). Sixty-one patients (56%) had radiographic abnormalities suggestive of active TB and such findings were more common among patients with confirmed or possible TB compared to those without TB (93%, 71%, vs. 47%; p = 0.002). Most patients (n = 91, 83%) had at least one radiographic abnormality—no difference by current TB classification was observed (93%, 100%, 79%; p = 0.08). The PPV of any current respiratory symptom, active TB radiographic finding, or any radiographic abnormality for TB was 13% (95%CI: 7–21%), 21% (95%CI: 12–34) and 14% (95%CI: 9–23), respectively; combining clinical and radiographic characteristics did not significantly improve the PPV for active TB.

**Conclusions:**

Among presumptive TB patients previously treated for TB, respiratory symptoms and radiographic abnormalities were common and poorly differentiated those with current active TB from those without current active TB. Reliance on clinical and radiographic characteristics alone in this patient population may result in substantial overtreatment and therefore, microbiological investigations should be used to inform TB treatment decisions whenever possible.

## Introduction

Due to active TB case finding strategies, improved diagnostics and more patient-centered treatment approaches, the treatment of drug-susceptible tuberculosis (TB) has become highly effective [1]. This has contributed to 58 million lives saved between 2000 and 2018 [2]. However, up to 65% TB survivors have some form of pulmonary impairment following microbiologic cure [3], termed post-TB lung disease (PTLD) [4]. Individuals with PTLD often have chronic respiratory symptoms, decreased functional capacity and persistent radiographic abnormalities. Unfortunately, PTLD is largely under-appreciated and under-addressed and contributes to a decreased quality of life and reduced economic productivity among afflicted TB survivors.

Given overlapping clinical and radiographic findings (e.g., poor specificity), as well as the limited access to extensive diagnostic tools, and shortage of expertise, in many high TB, low-resource settings, individuals with PTLD may be empirically re-treated with anti-TB therapy [5]. Not only does such misdiagnosis fail to address their underlying disease process—contributing to ongoing suffering—but it unnecessarily exposes them to potential adverse drug reactions. It also contributes to unnecessary health-care costs within busy and under-resourced TB programs.

Clinical mismanagement of individuals with PTLD in part reflects large gaps in knowledge regarding the clinical epidemiology of PTLD, especially among people living with HIV (PLHIV) [6]. In this study, we sought to characterize clinical and radiographic features among previously treated, presumptive TB patients presenting for evaluation in Zambia, and to determine whether these differed according to their current TB disease status. We also sought to determine whether clinico-radiological characteristics among presumptive TB patients without evidence of current TB—potentially indicative of PTLD—differed according to HIV status.

## Methods

### Study design and participants

This study represents a sub analysis of a prospective cross-sectional study nested within a large implementation research project. It was conducted between July and December 2018 at a primary health care TB diagnostic center that serves an informal peri-urban settlement in Lusaka, Zambia [7]. George Township is a peri-urban unplanned township in Lusaka with an estimated population of over 160,000 that has a high prevalence of HIV and TB. All consecutive adults (>18 years old) attending a level-one hospital in George township of Lusaka, Zambia for any reason were screened for the presence of TB. Patients were considered to have ‘presumptive TB’ if any of following symptoms, regardless of duration, were present: cough, fever, night sweats, unintentional weight loss, chest pain or loss of appetite. The present analysis was restricted to ‘presumptive TB patients’ with a self-reported, prior history of treatment for active TB disease.

### Ethics

All participants provided written informed consent in their primary language. Ethical approval was obtained from the University of Zambia Biomedical Research Ethics Committee, with further regulatory approvals obtained from the Zambian National Health Research Authority and the Lusaka District Health Office.

### Procedures and samples

Demographic details were recorded and each participant underwent standardized symptom-screening and clinical examination. All participants also underwent digital chest radiography (Odelca-DR, Delft Imaging Systems, The Netherlands) regardless of presenting symptoms. A sputum sample was obtained from all participants for mycobacteriology. A venous blood sample was requested for CD4 count determination (if HIV-positive). Any participant without a recent HIV test, or an unknown status, was offered free HIV testing.

### Laboratory procedures

Sputum specimens were processed at the Centre for Infectious Disease Research in Zambia (CIDRZ) central laboratory according to standardized protocols and quality assurance procedures. After decontamination with NALC-NaOH, the centrifuged sputum deposit was tested using concentrated acid-fast bacilli (AFB) fluorescence microscopy, Xpert Ultra (Cepheid, Sunnyvale, CA, USA) and mycobacterial culture (mycobacteria growth indicator tubes and Lowenstein Jensen Media [Becton Dickinson, Sparks, Maryland, USA]). Positive culture isolates were identified as *Mycobacterium tuberculosis* using MGIT TBc identification test (Becton Dickinson). CD4 cell counts for HIV-positive participants were determined using routine laboratory services.

### Radiography procedures

All chest X-rays were read independently by two radiologists (M.N. and I.D.) who were blinded to a participant’s TB status. Radiographic findings were classified according to characteristics of active TB or previous TB using a standardized reporting template [8]. All participants were then classified as having ‘any radiographic abnormality’, and/or ‘any radiographic abnormality suggestive of active TB,’ and/or ‘any radiographic abnormality suggestive of previous TB.’ Any differences in radiographic interpretation were reread and if differences persisted, results were finalized according to the reading of the more senior, experienced radiologist.

### Definitions and analysis

All patients were classified into one of three mutually exclusive categories according to their current TB disease status: confirmed TB, possible TB, no TB. Patients were defined as having ‘confirmed TB’ if *M*. *tuberculosis* was detected by Xpert Ultra (excluding a trace positive result) or cultured from a sputum sample. ‘Possible TB’ was defined by a trace positive Xpert Ultra result or an AFB smear microscopy positive result on sputum in the absence of a positive Xpert Ultra or culture result, or a patient who was empirically treated for TB based upon clinical and/or radiographic characteristics. ‘No TB’ was defined by the absence of any positive microbiological TB testing or receipt of empirical TB therapy.

Four separate analyses were performed. The first compared clinical and radiographic characteristics among all patients with a prior history of TB, according to their current TB status (according to microbiological and clinical criteria). The second analysis was a multivariable logistic regression, which sought to identify factors independently associated with active TB among presumptive patients previously treated for TB. This analysis reclassified those with ‘possible TB’ as having ‘no TB’ to create a binary variable (e.g., TB or no TB). Two multivariable models were constructed. For the first model, covariates in the univariable model meeting a predetermined P-value cutoff of ≤0.2, as well as a priori risk factors (age, sex, HIV status) were included in the multivariable model. For the second, a backward stepwise elimination model removed variables not meeting the predefined P-value exit criterion of ≤0.2 in the multivariable model. The third analysis sought to determine the diagnostic accuracy of manually-read chest X-rays among previously, treated presumptive TB patients—this included determining the sensitivity, specificity, positive predictive value (PPV) and negative predictive value (NPV). As with the above, this analysis reclassified those with ‘possible TB’ as having ‘no TB’ to create a binary variable (e.g., TB or no TB). An additional sensitivity analysis was undertaken to determine the diagnostic accuracy restricted to among patients with or without microbiological evidence of TB (e.g., possible TB patients excluded). To better contextualize the predictive value of chest X-rays for active TB, we also assessed the PPV of clinical symptoms and radiographic findings, both in isolation and in combination; combinations focused on what may be feasible to implement in limited-resource settings. The fourth analysis was restricted to among those without evidence of current TB (confirmed or possible) and compared clinical and radiographic characteristics according to HIV status; this analysis was undertaken to better understand whether characteristics of possible PTLD may differ according to HIV status.

As this was a secondary analysis of existing data and included all presumptive TB patients that were previously treated for TB, no formal sample size calculations or power analyses were undertaken [9]. Pearson’s chi-squared test and Fisher’s exact test were used to compared proportions as appropriate, while Wilcoxon rank-sum tests were used to compare median values. All statistical analyses were performed using STATA version 17.0.

## Results

### Patient characteristics

**Fig 1:**
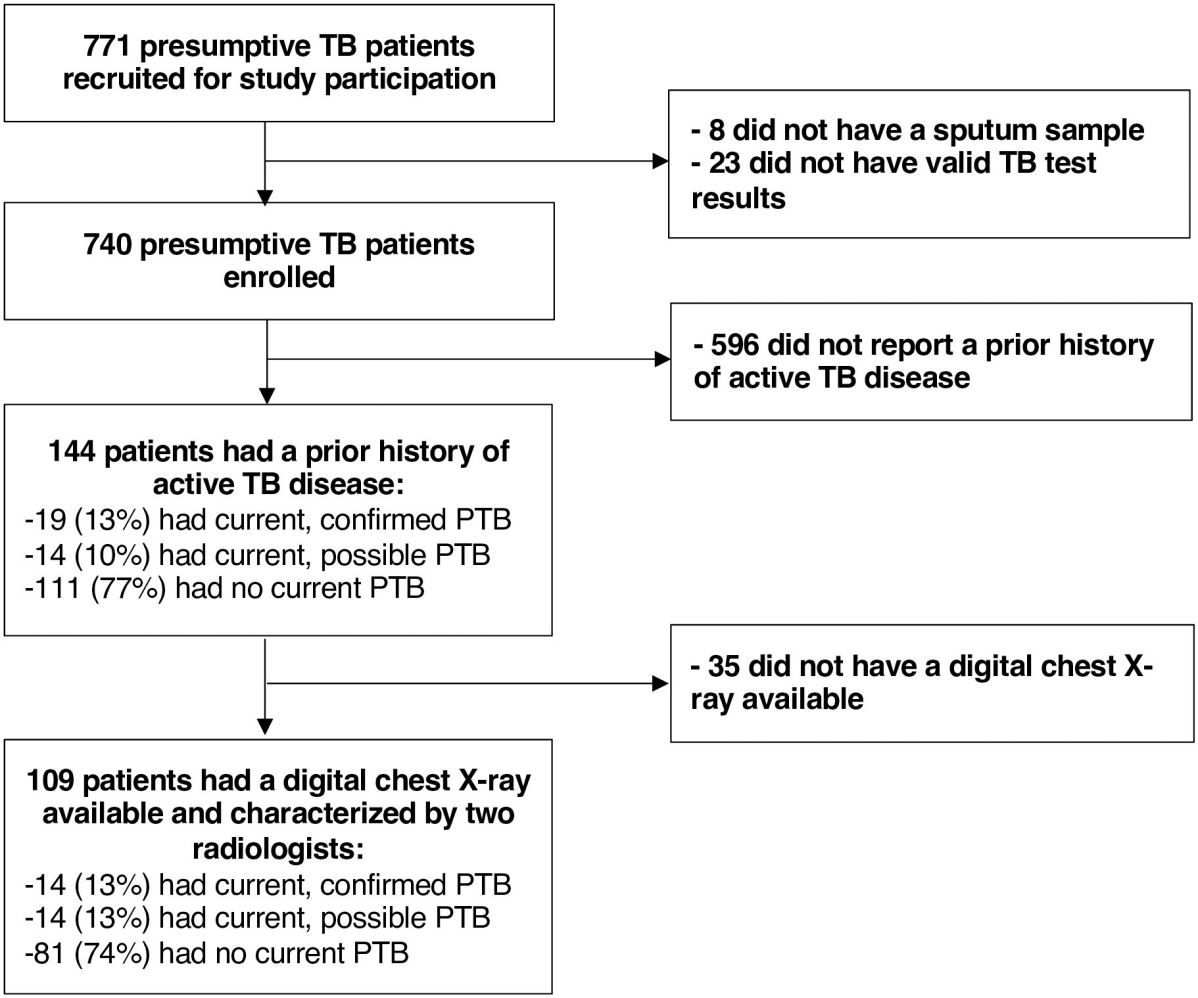
Study flow diagram.

Of 740 adults with presumptive TB who underwent microbiological testing and had valid results available, 144 (19%) had a prior history of being treated for active TB disease as well as complete TB microbiological test results available and were included (Fig 1). The median age among participants was 42 years, 69% of participants were male and 67% were HIV-positive (median CD4 count 263/μL) (Table 1). Overall, 19 of 144 (prevalence, 13% [95%CI: 8–20]) patients had confirmed TB, 14 (10%) patients had possible TB, and 111 (77%) patients had no current TB diagnosis; this was similar to the prevalence of confirmed TB among the 596 persons without a prior history of TB treatment (prevalence, 13%% [95%CI: 11–16]).

**Table 1:** Overview of baseline characteristics among presumptive TB patients with a prior history of TB, according to their current TB disease classification (n = 144).

	All (n = 144)	Confirmed TB (n = 19)	Possible TB (n = 14)	No TB (n = 111)	P-value
**Demographics**					
Age	42 (35–50)	37 (26–45)	46 (34–49)	42 (36–51)	0.32
Male	99 (68.8)	16 (84.2)	12 (85.7)	71 (64.0)	0.08
**Smoking status**					
Never	78 (54.2)	10 (52.6)	8 (57.1)	60 (54.1)	0.83
Former	32 (22.2)	6 (31.6)	3 (21.4)	23 (20.7)	
Current	34 (23.6)	3 (15.8)	3 (21.4)	28 (25.2)	
**HIV status**					
Positive	97 (67.4)	10 (52.6)	8 (57.1)	79 (71.2)	0.20
Negative	47 (32.6)	9 (47.4)	6 (42.9)	32 (28.8)	
**CD4 count If HIV-positive, median (IQR)** ^a^	263 (152–440)	250 (56–276)	225 (114–489)	270 (156–431)	0.62
**Symptoms**					
Cough (any duration)	95 (66.0)	17 (89.5)	12 (85.7)	66 (59.5)	0.01
Shortness of breath	57 (39.6)	10 (52.6)	9 (64.3)	38 (34.2)	0.047
Chest pain	97 (67.4)	13 (68.4)	12 (85.7)	72 (64.9)	0.32
Any respiratory symptom	119 (82.6)	18 (94.7)	13 (92.9)	88 (79.3)	0.23
WHO HIV-TB Symptom screen	106 (73.6)	17 (89.5)	13 (92.9)	76 (68.5)	0.043
Fevers	42 (29.2)	8 (42.1)	3 (21.4)	31 (27.9)	0.37
Weight loss	61 (42.4)	12 (63.2)	7 (50.0)	42 (37.8)	0.10
Night sweats	37 (25.7)	6 (31.6)	5 (35.7)	26 (23.4)	0.45
**Physical exam**					
Respiratory rate^b^	20 (18–22)	20 (18–22)	20 (18–20)	20 (18–22)	0.56
Pulse^c^	92 (78–102)	101 (87–119)	98 (67–110)	89 (78–101)	0.05
O_2_ Saturation	98 (97–100)	98 (94–100)	99 (98–100)	98 (97–100)	0.36
Body mass index^d^	19 (17–22)	17 (15–19)	20 (19–21)	19 (17–22)	0.028
Abnormal chest auscultation^e^	15 (10.8)	5 (29.4)	5 (35.7)	5 (4.6)	<0.001
Lymphadenopathy^e^	4 (2.9)	2 (11.8)	0	2 (1.9)	0.12

*Any respiratory symptom defined as the presence of cough, shortness of breath or dyspnea.

^a^4 missing values,

^b^1 missing value,

^c^2 missing values,

^e^5 missing values.

Details of the 14 possible TB cases among those previously treated for TB are summarized in S1 Table. Notably, 13/14 of patients with possible TB were started on anti-TB therapy of whom 7/13 had a trace-positive Xpert Ultra result; thus, 6/117 (5%; 6/14 patients with possible TB and 0/111 patients with no TB) of all patients without any microbiological evidence of pulmonary TB received empirical anti-TB therapy.

### Patient characteristics according to current TB status

Previously treated, presumptive TB patients did not differ by age, sex, HIV status, smoking status according to their current TB disease classification (Table 1). The large majority (83%) of patients reported at least one, current respiratory symptom (cough, dyspnea or chest pain) but this did not differ by current TB disease status (95%, 93% vs. 79%. p = 0.23). Confirmed and possible TB patients were however, more likely to report current cough (90%, 86%, vs. 60%; p = 0.01) and dyspnea (53%, 64%, vs. 34%; p = 0.05), but not chest pain (68%, 86%, vs. 65%; p = 0.32) compared to patients without TB. Most patients (74%) also had a positive WHO symptom screen; while confirmed TB (90%) and possible TB patients (93%) were more likely to have a positive WHO symptom screen, 69% of patients without TB also screened positive. There were few additional differences with regard to clinical symptoms and signs according to current TB classification (Table 1).

### Radiography characteristics according to current TB status

Overall, 61 (56%) previously treated, presumptive TB patients had radiographs with at least one finding suggestive of active TB. Compared to patients without TB, those with confirmed TB (93%) or possible TB (71%) were more likely to have radiographic features consistent with active TB disease (p = 0.002); however, almost half (47%) of patients without evidence of pulmonary TB had at least one radiographic finding suggestive of active TB disease (Table 2). While individual radiographic findings suggestive of active TB, including cavitation, focal consolidation and reticular interstitial pattern were more common among those with confirmed and possible TB, such findings were also frequently identified among those with no evidence of current pulmonary TB (Table 2).

**Table 2:** Overview of chest radiography characteristics among presumptive TB patients with a prior history of TB, according to their current TB disease classification (n = 109).

	Overall (n = 109)	Confirmed TB (n = 14)	Possible TB (n = 14)	No TB (n = 81)	P-value
**Findings suggestive of active TB**					
Cavitation	32 (29.4)	8 (57.1)	7 (50.0)	17 (21.0)	0.004
Focal consolidation	30 (27.5)	10 (71.4)	6 (42.9)	14 (17.3)	<0.001
Reticular interstitial pattern	42 (38.5)	10 (71.4)	6 (42.9)	26 (32.1)	0.020
Miliary nodules	1 (0.9)	1 (7.1)	0	0	0.26
Lymphadenopathy	19 (17.4)	2 (14.3)	3 (21.4)	14 (17.3)	0.92
Pleural effusion/empyema	18 (16.5)	4 (28.6)	3 (21.4)	11 (13.6)	0.29
Any abnormality suggestive of active TB	61 (56.0)	13 (92.9)	10 (71.4)	38 (46.9)	0.002
**Findings suggestive of prior TB**					
Peri-bronchial fibrosis	56 (51.4)	9 (64.3)	9 (64.3)	38 (46.9)	0.31
Nodular opacities	41 (37.6)	9 (64.3)	5 (35.7)	27 (33.3)	0.09
Traction bronchiectasis	44 (40.4)	8 (57.1)	7 (50.0)	29 (35.8)	0.22
Apical and upper lobe volume loss	58 (53.2)	10 (71.4)	9 (64.3)	39 (48.2)	0.21
Calcified granulomas and/or lymph nodes	21 (19.3)	6 (42.9)	4 (28.6)	11 (13.6)	0.021
Pleural thickening and/or calcification	63 (57.8)	11 (78.6)	10 (71.4)	42 (51.9)	0.11
Any abnormality suggestive of prior TB	81 (74.3)	12 (85.7)	11 (78.6)	58 (71.6)	0.50
Any abnormality suggestive of TB (active or prior)	91 (83.5)	13 (92.9)	14 (100)	64 (79.0)	0.08

Most patients (74%) had at least one radiographic abnormality consistent with prior TB disease, however, this did not differ according to current TB classification (86%, 79%, 72%; p = 0.50). Similarly, the presence of any radiographic abnormality (active or prior TB) was very common overall (84%) and also did not differ according to current TB classification (93%, 100%, 79%; p = 0.08).

### Predictors of active TB among previously treated presumptive TB patients

Multivariable logistic regression analysis was undertaken to identify predictors of active TB (Table 3). Several variables demonstrated an association with active TB in univariable analysis; however, in both multivariable analyses, the only independent predictor was a chest X-ray finding suggestive of active TB, which demonstrated a strong association with confirmed pulmonary TB (Table 3).

**Table 3:** Unadjusted and adjusted odds ratio for active tuberculosis among previously treated, presumptive TB patients (n = 100).

	Unadjusted Odds ratio (95%CI)	P-value	Model #1, Adjusted Odds ratio (95%CI)	P-value	Model #2, Adjusted Odds ratio (95%CI)	P-value*
**Age (for each year increase)**	0.96 (0.92–1.01)	0.10	0.97 (0.91–1.02)	0.32		
**Male sex**	2.70 (0.74–9.78)	0.10	1.77 (0.28–11.41)	0.53		
**Smoking status**						
Never	1	0.49				
Former	0.66 (0.17–2.56)					
Current	1.66 (0.08–0.29)					
**HIV status**						
Negative	1	0.15	1	0.56		
Positive	2.06 (0.77–5.48)		1.56 (0.35–6.93)			
**Symptoms**						
Cough	5.12 (1.13–23.17)	0.012				
Shortness of breath	1.84 (0.70–4.87)	0.22				
Chest pain	1.06 (0.37–2.98)	0.92				
Fevers	1.95 (0.72–5.25)	0.20				
Weight loss	2.66 (0.98–7.22)	0.05				
Night sweats	1.40 (0.49–4.00)	0.54				
Number of WHO symptoms (for each 1 unit increase)^#^	1.50 (1.04–2.16)	0.03	0.90 (0.52–1.57)	0.71		
**Signs**						
Respiratory rate (for each unit increase)	2.00 (0.68–5.88)	0.19	1.13 (0.17–7.51)	0.90		
Pulse (for each unit increase)	1.05 (1.01–1.09)	0.005	1.04 (0.98–1.10)	0.15	1.03 (0.98–1.08)	0.11
O2 Saturation (for each unit increase)	0.91 (0.82–1.02)	0.12	1.09 (0.84–1.41)	0.52		
Body mass index (for each unit decrease)	0.81 (0.67–0.97)	0.023	0.82 (0.62–1.08)	0.12	0.82 (0.64–1.06)	0.16
**Chest radiography**						
Any CXR abnormality suggestive of active TB	12.72 (1.60–101.21)	0.016	7.98 (0.84–75.51)	0.031	8.76 (1.03–74.53)	0.013
Any CXR abnormality suggestive of TB (active or prior)	2.83 (0.35–23.15)	0.33				

For Model #1 covariates in the univariable model meeting a predetermined P-value cutoff of ≤0.2, as well as a priori risk factors (age, sex, HIV status) were included in the multivariable model. For Model #2, a backward stepwise elimination model removed variables not meeting the predefined P-value exit criterion of ≤0.2 in the multivariable model. Model #1 included ‘number of WHO symptoms;’ individual clinical symptoms meeting the predefined cutoff for inclusion in the multivariable model were cough, weight loss and fever of any duration. However, such variables were each accounted for within the composite symptom variable (‘number of WHO symptoms’) variable. When these 3 individual variables were included in Model #1 instead of the composite symptom variable, the overall effect sizes of other variables were unchanged and none of the three clinical symptoms achieved significance at the level of p<0.1.

^#^Symptoms include cough, weight loss, fever. and night sweats of any duration.

P-values represent likelihood ratio test.

### Diagnostic utility of chest radiograph findings among previously treated presumptive TB patients

**Fig 2:**
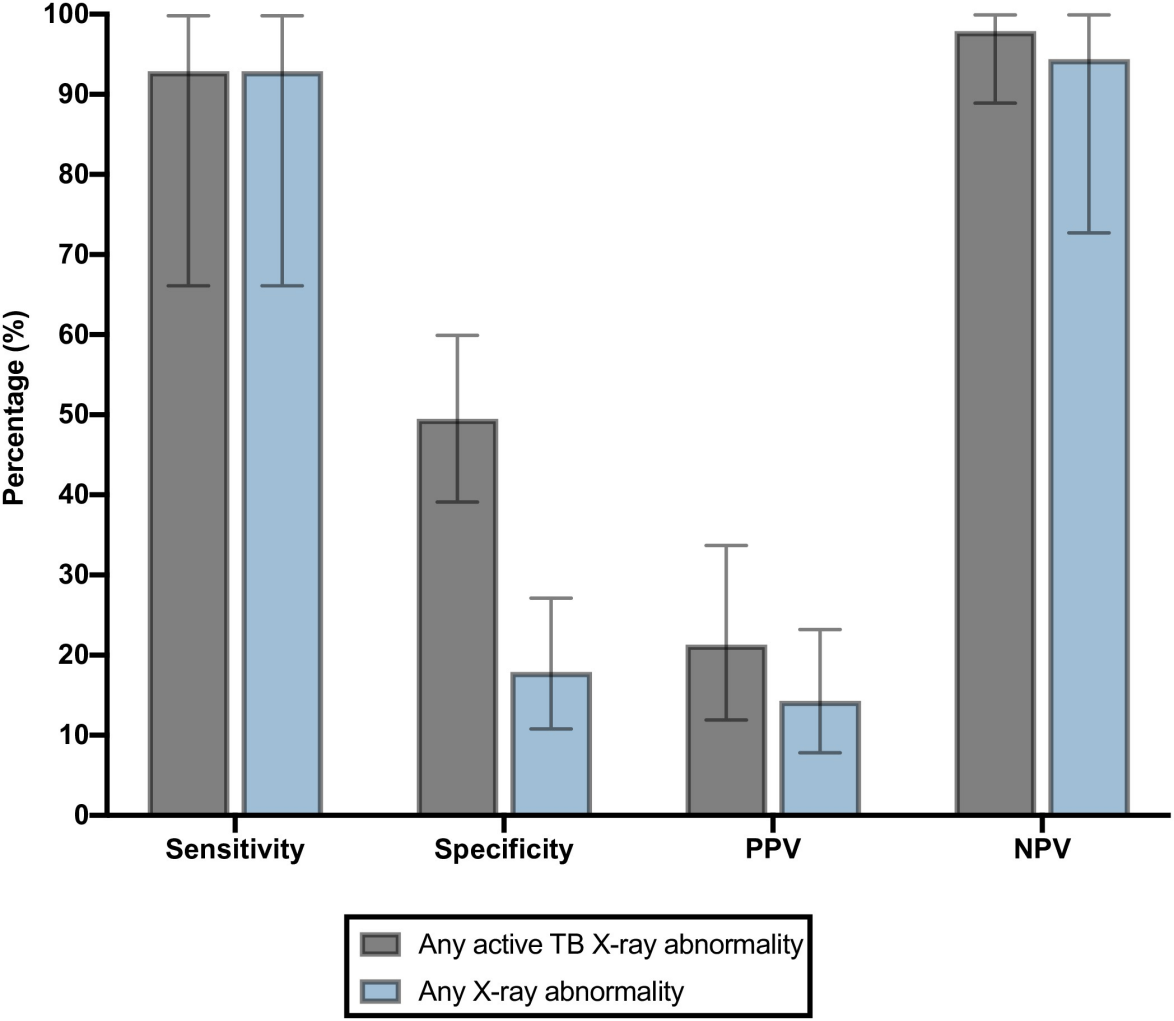
Diagnostic accuracy of chest radiograph findings for active TB disease among presumptive TB patients with a prior history of TB disease (n = 109). Bars refer to 95% confidence intervals. Abbreviations: PPV = positive predictive; NPV = negative predictive value.

Next, we sought to determine the diagnostic accuracy of manually-read chest X-rays for current pulmonary TB disease among presumptive TB patients with a prior TB treatment history. The sensitivity, specificity, PPV and NPV of any radiographic abnormality suggestive of active TB for microbiologically-confirmed TB was 93%, 50%, 21% and 98%, respectively (Fig 2). When a positive result was defined by any radiographic abnormality (possibly active or prior TB features), the sensitivity, specificity, PPV and NPV were 93%, 18%,14% and 94%, respectively (Fig 2). In a sensitivity analysis that excluded those classified as having ‘possible TB’, diagnostic accuracy estimates were similar (S2 Table).

To better contextualize the PPV of chest X-rays in presumptive TB patients with a prior TB treatment history, we evaluated the PPVs of individual clinical symptoms and radiographic characteristics as well as in combination (Table 4). Except for military nodules, which were present in only a single patient, symptoms and radiographic findings had low PPV for active pulmonary TB. PPV was highest in those with 4/4 WHO symptoms and those with a focal consolidation on chest radiograph, but only reached 33% for these characteristics. Combining clinical symptoms and chest X-ray findings did not significantly improve PPV (Table 4).

**Table 4:** The positive predictive of clinical symptoms and chest radiography findings among presumptive TB patients previously treated for TB (n = 109).

	Number with characteristic	PPV (95% CI)
**Symptoms (individual)**		
Cough (any duration)	78/109	15.4 (8.2–25.3)
Shortness of breath	49/109	16.3 (7.3–29.7)
Chest pain	82/109	12.2 (6.0–21.3)
Fevers	31/109	16.1 (5.5–33.7)
Weight loss	46/109	15.2 (6.3–28.9)
Night sweats		
**Symptoms (combination)**		
Any respiratory symptom*	100/109	13.0 (7.1–21.2)
≥2 respiratory symptoms	73/109	15.1 (7.8–25.4)
3 respiratory symptoms	36/109	16.7 (6.4–32.8)
Any WHO screen symptom**	86/109	14.0 (7.4–23.1)
≥2 symptoms	57/109	12.3 (5.1–23.7)
≥3 symptoms	32/109	15.6 (5.3–32.8)
4 symptoms	12/109	33.3 (9.9–65.1)
**Radiographic findings suggestive of active TB (individual)**		
Cavitation	32/109	25.0 (11.5–43.4)
Focal consolidation	30/109	33.3 (17.3–52.8)
Reticular interstitial pattern	32/109	23.8 (12.1–39.5)
Miliary nodules	1/109	100 (2.5–100)
Lymphadenopathy	19/109	10.5 (1.3–33.1)
Pleural effusion/empyema	18/109	22.2 (6.4–47.6)
**Radiographic findings suggestive of prior TB (individual)**		
Peri-bronchial fibrosis	56/109	16.1 (7.6–28.3)
Nodular opacities	41/109	22.0 (10.6–37.6)
Traction bronchiectasis	44/109	18.2 (8.2–32.7)
Apical and upper lobe volume loss	58/109	17.2 (8.6–29.4)
Calcified granulomas and/or lymph nodes	21/109	28.6 (11.3–52.2)
Pleural thickening and/or calcification	63/109	17.5 (9.1–29.1)
**Radiographic findings (combination)**		
Any abnormality suggestive of active TB	61/109	21.3 (11.9–33.7)
Any abnormality suggestive of prior TB	81/109	14.8 (7.9–24.4)
Any CXR abnormality	91/109	14.3 (7.8–23.2)
**Algorithms**		
Cough (any duration) and any active TB CXR abnormality	50/109	24.0 (13.1–38.2)
Cough (any duration) and any CXR abnormality	67/109	17.9 (9.6–29.2)
Any respiratory symptom* and any active TB CXR abnormality	58/109	22.4 (12.5–35.3)
Any respiratory symptom* and any CXR abnormality	85/109	15.3 (8.4–24.7)
WHO screen positive and any active TB CXR abnormality	52/109	23.1 (12.5–36.8)
WHO screen positive and any CXR abnormality	74/109	16.2 (8.7–26.6)

*Cough, shortness of breath or chest pain (any duration).

*Fevers, cough, night sweats, unintentional weight loss (any duration).

Abbreviations: CXR = chest X-ray; PPV = positive predictive value; TB = tuberculosis.

### Clinical and radiographic characteristics in presumptive TB patients without evidence of current TB according to HIV status

Of 111 presumptive TB patients without any evidence of current pulmonary TB (possibly indicative of PTLD), 79 (71%) were HIV-positive and 32 (29%) were HIV-negative. HIV-negative patients were more likely to be male and to be a current or former smoker (S3 Table). Overall, clinical symptoms and signs did not differ according to HIV status, with the exception that HIV-negative patients were more likely to have at least one current respiratory symptom (94% vs. 73%, p = 0.019) and current night sweats (38% vs. 18%, p = 0.026) compared to HIV-positive patients (S3 Table).

Among presumptive TB patients without any evidence of pulmonary TB, a large proportion (47%) had at least one radiographic finding suggestive of active TB (S4 Table); while this did not differ according to HIV status, there was a trend towards active TB radiographic abnormalities being more common among HIV-negative compared to HIV-positive patients (60% vs. 41%; p = 0.12). With the exception of lymphadenopathy which demonstrated a trend towards being more common among HIV-negative patients compared to HIV-positive patients (28% vs. 13%; p = 0.09), specific active TB radiographic abnormalities did not differ according to HIV status.

Radiographic abnormalities suggestive of prior TB were common in both groups and while they appeared to be more common among HIV-negative patients compared to HIV-patients, this did not reach statistical significance (83% vs. 66%; p = 0.12, S4 Table). HIV-negative patients were however much more likely to have evidence of peri-bronchial fibrosis compared to HIV-positive patients (68% vs. 38%; p = 0.011). Additionally, there was a trend towards HIV-negative patients being more likely to have apical volume loss compared to HIV-negative patients (64% vs. 41%; p = 0.06). No additional differences by HIV status with respect to individual radiographic characteristics suggestive of prior TB disease were noted. The overall prevalence of any radiographic abnormality was also similar, irrespective of HIV status (75% vs. 88%; p = 0.18).

## Discussion

In this study conducted in Zambia among individuals previously treated for TB who were presenting for evaluation of a possible TB illness, more than 20% had either confirmed or possible pulmonary TB disease. However, the majority of such patients had respiratory symptoms and radiographic abnormalities regardless of whether or not they had current TB disease—thus, such findings were non-specific and had a low positive predictive value for current pulmonary TB disease. These results have important implications for other high TB burden settings in which access to timely TB microbiological testing and/or results may be limited. In such settings there may be a reliance on clinical and radiographic characteristics alone to inform treatment decisions despite recommendations that they should only be used for screening and triage purposes [10]. For example, in Zambia almost half of the 37,000 persons with pulmonary TB who were initiated on anti-TB therapy in 2020 were clinically diagnosed [11].

Nearly one-in-five individuals presenting for evaluation at a public health facility with presumptive TB had previously been treated for active TB. This is consistent with findings from other high burden settings in Africa and Asia that demonstrate that a large proportion of presumptive TB patients have a prior TB treatment history [12–14]. As patient-centered strategies to improve TB treatment [15], including shorter-duration regimens [16], are implemented, the proportion of patients successfully cured of TB will continue to increase and in turn, so too will the number of TB survivors. Many of these persons will go on to have PTLD marked by chronic pulmonary dysfunction and resultant decreased quality of life. For example, a recent study conducted in Malawi, a high TB/HIV setting, found that after a single, successfully treated pulmonary TB episode, one-third of individuals had abnormal spirometry, 40% had bronchiectasis, and 31% reported chronic respiratory symptoms after one year that often affected their work [17]. Such persons however, also remain at higher risk for subsequent episodes of active TB disease and are more likely to have multi-drug resistant TB strain [18]. TB survivors with PTLD are likely to constitute an increasingly large proportion of presumptive TB patients seeking care with respiratory symptoms. Therefore, ongoing research is urgently needed to (1) understand the best tools and strategies to rapidly differentiate PTLD from recurrent TB and other respiratory diseases, and (2) determine the optimal support and treatment strategies for TB survivors living with PTLD.

In our study, conducted under routine program conditions, we found that at least 5% of symptomatic individuals, previously treated for TB, were once again treated for active TB disease despite negative sputum culture and Xpert Ultra results; this was likely on the basis of clinical presentation combined with abnormal chest radiography. While empiric therapy for 1 in 20 previously treated, presumptive TB patients may at first glance appear low, at scale within large TB treatment programs, this could result in inappropriate treatment of hundreds to thousands of patients annually. A prior study from Zimbabwe similarly found that individuals previously treated for TB constituted a large proportion of patients empirically treated for TB despite extensive negative microbiological testing—nearly 75% of these patients had abnormal radiographs consistent with prior TB [5]. While further epidemiological data is lacking, empirical TB therapy is likely to be even more common in high TB burden settings where access to rapid TB diagnostics may be limited or unreliable and/or there is limited knowledge about PTLD and its clinical and radiographic manifestations.

Notably, we found that TB symptoms, including respiratory symptoms and abnormal chest radiography were extremely common among previously treated presumptive patients regardless of current TB disease classification and that without the use of adjunctive confirmatory microbiological TB tests, would be unhelpful for differentiating between patients with and without current active TB. In multivariable analyses clinical symptoms and signs were not predictive of current TB disease. While an abnormal chest radiograph suggestive of TB was independently associated with an approximately 8-times higher odds of active TB, this finding only had a PPV of 21%—reflecting the known poor specificity associated with chest X-rays [10]. Additionally, the PPV of specific chest X-ray findings alone or in combination for active TB was low and never exceeded 33% among previously treated, presumptive TB patients. These results suggest that while such patients have a high risk of active TB disease, they also imply that at best, reliance on clinical symptoms and chest radiograph findings without the use of confirmatory tests for TB would result in at least 2 patients without TB being inappropriately treated for TB for every 1 patient who has TB. Further, these findings were despite radiographs being read under ideal conditions by two highly experienced radiologists, which likely overestimates their performance under routine programmatic conditions. Our results strongly suggest that whenever possible, retreatment of presumptive TB patients should be guided by positive microbiological test results in order to avoid unnecessary drug toxicity due to anti-TB therapy, and also not delay appropriate diagnosis and treatment of an alternative etiology that may be underpinning their presentation. For example, for persons with PTLD with persistent respiratory symptoms and functional impairment but without current TB disease, appropriate treatment includes outpatient pulmonary rehabilitation [19], which can improve the quality of life in TB survivors and possibly inhaled bronchodilators, which may improve dyspnea and prevent decline in lung function; however, definitive evidence on the efficacy of bronchodilators in this setting is not yet available [20].

There is limited knowledge regarding how PTLD among TB survivors may differ between HIV-positive and HIV-negative individuals. To address this knowledge gap, this study secondarily sought to assess whether clinical signs and symptoms as well as radiography differed according to HIV status among previously treated, presumptive TB patients without microbiological evidence of current pulmonary TB disease. We found very few clinical differences or radiographic differences in this patient sub-group beyond a slightly higher prevalence of current respiratory symptoms among HIV-negative individuals as well as a higher proportion of peri-bronchial fibrosis and apical volume loss among HIV-negative individuals. In the aforementioned study from Malawi, among patients who recently completed TB therapy, HIV-negative patients were more likely to have residual respiratory symptoms and abnormal chest tomography features, especially airway and parenchymal pathologies, but a high-burden of PLTD was present in both patient groups [17]. There is still an urgent need to undertake further prospective studies defining the epidemiology of PTLD in high TB/HIV settings as well as to define effective and sustainable treatment and support strategies that can be implemented in resource- limited settings for TB survivors with PTLD after discharge from routine TB treatment programs.

Strengths of this study include the fact that individuals with prior TB living in a high burden TB setting presenting for evaluation for any reason were systematically screened and tested for the presence of TB. Additionally, all X-rays were independently read by two experienced radiologists according to standardized criteria. There were however some limitations. While a large number of presumptive TB patients with prior TB were identified, the number of patients was not large enough to undertake robust subgroup analyses according to HIV status and multivariable analyses may have been underpowered; therefore, some true differences in clinical and radiographic characteristics may have been missed due to statistical underpowering. Furthermore, while we enrolled patients that were well-characterized, we did not have additional investigations such a sputum bacterial and fungal cultures, chest computed tomography (CT) imaging or pulmonary function tests that allowed us to elucidate the etiology underpinning respiratory symptoms among persons without radiographic or microbiological evidence of current pulmonary TB. Therefore, while it’s likely many of such patients had PTLD, we cannot be certain that they did not have alternative or multiple pulmonary processes. Finally, we had insufficient data available to compare the predictive value of manually read chest radiographs among those with a prior TB history to those without a prior TB history. The PPV of chest radiography for active TB among presumptive patients with a history of TB treatment in this study (21%) was lower than was previously found in Lusaka, Zambia among presumptive TB patients regardless of TB treatment history (33%) using chest radiography with computer-aided detection software [21]. In general, the performance of chest radiography as a screening and triage tool for TB disease according to prior TB treatment status is poorly characterized; however, a recent study from Bangladesh demonstrated that chest radiography using several different computer-aided detection software had substantially reduced diagnostic performance for active TB among those previously treated for TB [22]. Thus, it is important that future studies evaluate the accuracy of chest radiography and other TB screening and triage tools suitable for use in resource-limited settings (e.g., screening algorithms, prediction models, point-of-care tests), according to prior TB treatment status, to define optimal diagnostic strategies for this important patient population.

In conclusion, among presumptive TB patients with a prior history of TB disease, TB respiratory symptoms and radiographical abnormalities were commonly identified, but were non-specific and poorly differentiated active TB disease. Chest X-rays should be interpreted with caution among previously treated, presumptive TB patients and whenever possible, TB therapy decisions should be guided by microbiological TB investigations.

## Supporting information

S1 Table Characteristics of previously treated patients with ‘possible’ current TB (n = 14). (DOCX)

S2 Table Diagnostic accuracy of chest radiograph findings for active TB disease among presumptive TB patients with a prior history of TB disease. The first analysis reclassified those with ‘possible TB’ as having ‘no TB’ to create a binary variable (e.g., TB or no TB) (n = 109). The second analysis excluded those classified as having ‘possible TB’ (n = 95).
(DOCX)

S3 Table Overview of baseline characteristics among presumptive TB patients with a prior history of TB, without evidence of current TB disease (confirmed or possible), according to HIV status (n = 111). (DOCX)

S4 Table Overview of chest radiography characteristics among presumptive TB patients with a prior history of TB, without evidence of current TB disease (confirmed or possible), according to HIV status (n = 81). (DOCX)

S1 Data (XLSX)
