# Supplementary material for: Clinical and radiographic characteristics of presumptive tuberculosis patients previously treated for tuberculosis in Zambia
Source: PLoS One. 2022 Jan 27;17(1):e0263116. doi: 10.1371/journal.pone.0263116 (PMC8794156; doi:10.1371/journal.pone.0263116)
Supplement: S1 Table — (DOCX) [file pone.0263116.s001.docx]

**S1 Table. Characteristics of previously treated patients with ‘possible’ current TB (n=14).**

| **Patient** | **Age** | **Sex** | **HIV status**  **(CD4 count)** | **Symptoms** | **Active TB features on Chest X-ray?** | **Sputum smear microscopy**  **result** | **Sputum culture result** | **Sputum Xpert result** | **Treated for TB?** |
| --- | --- | --- | --- | --- | --- | --- | --- | --- | --- |
| **1** | 45 | M | Neg. | Cough, weight loss, chest pain, shortness of breath | Yes | Neg. | Neg. | Neg. | Yes |
| **2** | 31 | M | Neg. | Cough, night sweats, chest pain, shortness of breath | Yes | Neg. | Neg. | Neg. | Yes |
| **3** | 49 | M | Neg. | Cough, chest pain | Yes | Neg. | Neg. | Trace Pos. | Yes |
| **4** | 58 | M | Neg. | Cough, weight loss, night sweats, chest pain, shortness of breath, fevers | Yes | Neg. | Neg. | Neg. | Yes |
| **5** | 50 | M | Pos (114) | Cough, chest pain, shortness of breath | Yes | Neg. | Neg. | Neg. | Yes |
| **6** | 46 | M | Neg. | Chest pain, shortness of breath | No | Neg. | Neg. | Neg. | Yes |
| **7** | 34 | M | Pos (N/A) | Cough, weight loss, chest pain, shortness of breath, fevers | Yes | Neg. | Neg. | Neg. | Yes |
| **8** | 26 | M | Pos (489) | Cough, weight loss, chest pain | Yes | Neg. | Neg. | Trace Pos. | Yes |
| **9** | 53 | M | Pos (606) | Cough, weight loss, night sweats, chest pain, shortness of breath | Yes | Neg. | Neg. | Trace Pos. | Yes |
| **10** | 35 | F | Pos (225) | Cough, weight loss, chest pain | Yes | Neg. | Neg. | Trace Pos. | Yes |
| **11** | 31 | M | Pos (348) | Cough, night sweats, shortness of breath | Yes | Neg. | Neg. | Trace Pos. | Yes |
| **12** | 40 | F | Neg. | Cough, chest pain | No | Neg. | Neg. | Trace Pos. | Yes |
| **13** | 47 | M | Pos (55) | Cough, night sweats, chest pain, shortness of breath | No | Neg. | Neg. | Trace Pos. | Yes |
| **14** | 46 | M | Pos (173) | Weight loss, fevers | No | Neg. | Neg. | Trace Pos. | No |

Abbreviations: ‘F’=female; ‘M’=male; ‘pos’=positive; ‘neg’=negative
