# Supplementary material for: Clinical and radiographic characteristics of presumptive tuberculosis patients previously treated for tuberculosis in Zambia
Source: PLoS One. 2022 Jan 27;17(1):e0263116. doi: 10.1371/journal.pone.0263116 (PMC8794156; doi:10.1371/journal.pone.0263116)
Supplement: S2 Table — The first analysis reclassified those with ‘possible TB’ as having ‘no TB’ to create a binary variable (e.g., TB or no TB) (n = 109). The second analysis excluded those classified as having ‘possible TB’ (n = 95). (DOCX) [file pone.0263116.s002.docx]

**S2 Table. Diagnostic accuracy of chest radiograph findings for active TB disease among presumptive TB patients with a prior history of TB disease.** The first analysis reclassified those with ‘possible TB’ as having ‘no TB’ to create a binary variable (e.g., TB or no TB) (n=109). The second analysis excluded those classified as having ‘possible TB’ (n=95).

|  | **n/N** | **Sensitivity**  **(95% CI)** | **n/N** | **Specificity**  **(95% CI)** | **n/N** | **PPV**  **(95% CI)** | **n/N** | **NPV**  **(95% CI)** |
| --- | --- | --- | --- | --- | --- | --- | --- | --- |
| **Analysis #1 (n=109)** | | | | | | | | |
| **Positive case defined by any active TB abnormality** | 13/14 | 92.9 (66.1-99.8) | 47/95 | 49.5 (39.1-59.9) | 13/61 | 21.3 (11.9-33.7) | 47/48 | 97.9 (88.9-99.9) |
| **Positive case defined by any chest X-ray abnormality** | 13/14 | 92.9 (66.1-99.8) | 17/95 | 17.9 (10.8-27.1) | 13/91 | 14.3 (7.8-23.2) | 17/18 | 94.4 (72.7-99.9) |
| **Analysis #2 (n=95)** | | | | | | | | |
| **Positive case defined by any active TB abnormality** | 13/14 | 92.9 (66.1-99.8) | 43/81 | 53.1 (41.7-64.3) | 13/51 | 25.5 (14.3-39.6) | 43/44 | 97.7 (88.0-99.9) |
| **Positive case defined by any chest X-ray abnormality** | 13/14 | 92.9 (66.1-99.8) | 17/81 | 21.0 (12.7-31.5) | 13/77 | 16.9 (9.3-27.1) | 17/18 | 94.4 (72.7-99.9) |
