# Supplementary material for: Clinical and radiographic characteristics of presumptive tuberculosis patients previously treated for tuberculosis in Zambia
Source: PLoS One. 2022 Jan 27;17(1):e0263116. doi: 10.1371/journal.pone.0263116 (PMC8794156; doi:10.1371/journal.pone.0263116)
Supplement: S3 Table — (DOCX) [file pone.0263116.s003.docx]

**S3 Table. Overview of baseline characteristics among presumptive TB patients with a prior history of TB, without evidence of current TB disease (confirmed or possible), according to HIV status (n=111).**

|  | **All**  **(n=111)** | **HIV-positive**  **(n=79)** | **HIV-negative**  **(n=32)** | **P-value** |
| --- | --- | --- | --- | --- |
| **Demographics** |  |  |  |  |
| Age | 42 (36-51) | 42 (36-51) | 42 (37-46) | 0.52 |
| Male | 71 (64.0) | 44 (55.7) | 27 (84.4) | 0.004 |
| **Smoking status** |  |  |  |  |
| Never | 60 (54.1) | 49 (62.0) | 11 (34.4) | 0.028 |
| Former | 23 (20.7) | 13 (16.5) | 10 (31.3) |  |
| Current | 28 (25.2) | 17 (21.5) | 11 (34.4) |  |
| **CD4 count If HIV-positive, median (IQR)^a^** | 270 (156-431) | 270 (156-431) | - | - |
| **Symptoms** |  |  |  |  |
| Cough (any) | 66 (59.5) | 46 (58.2) | 20 (62.5) | 0.68 |
| Productive cough | 3 (2.7) | 1 (1.3) | 2 (6.3) | 0.20 |
| Shortness of breath | 38 (34.2) | 24 (30.4) | 14 (43.8) | 0.18 |
| Chest pain | 72 (64.9) | 47 (59.5) | 25 (78.1) | 0.06 |
| Any respiratory symptom (cough, SOB, chest pain) | 88 (79.3) | 58 (73.4) | 30 (93.8) | 0.019 |
| WHO HIV-TB Symptom screen | 76 (68.5) | 52 (65.8) | 24 (75.0) | 0.35 |
| Fevers | 31 (27.9) | 24 (30.4) | 7 (21.9) | 0.37 |
| Weight loss | 42 (37.8) | 30 (38.0) | 12 (37.5) | 0.96 |
| Night sweats | 26 (23.4) | 14 (17.7) | 12 (37.5) | 0.026 |
| **Physical exam** |  |  |  |  |
| Respiratory rate^b^ | 20 (18-22) | 20 (18-22) | 20 (18-22) | 0.69 |
| Pulse^b^ | 89 (78-101) | 87 (74-101) | 98 (82-103) | 0.08 |
| O2 Saturation | 98 (97-100) | 99 (97-100) | 98 (97-99) | 0.14 |
| Body mass index^c^ | 19 (17-22) | 19 (17-23) | 19 (17-20) | 0.40 |
| Abnormal chest auscultation^d^ | 5 (4.6) | 3 (3.9) | 2 (6.5) | 0.62 |
| Lymphadenopathy^d^ | 2 (1.9) | 2 (2.3) | 0 | 1 |

^a^2 missing values, ^b^1 missing values, ^c^7 missing values, ^d^3 missing values
