# Supplementary material for: Clinical and radiographic characteristics of presumptive tuberculosis patients previously treated for tuberculosis in Zambia
Source: PLoS One. 2022 Jan 27;17(1):e0263116. doi: 10.1371/journal.pone.0263116 (PMC8794156; doi:10.1371/journal.pone.0263116)
Supplement: S4 Table — (DOCX) [file pone.0263116.s004.docx]

**S4 Table**. **Overview of chest radiography characteristics among presumptive TB patients with a prior history of TB, without evidence of current TB disease (confirmed or possible), according to HIV status (n=81).**

|  | **All**  **(n=81)** | **HIV-positive**  **(n=56)** | **HIV-negative**  **(n=25)** | **P-value** |
| --- | --- | --- | --- | --- |
| **Findings suggestive of active TB** |  |  |  |  |
| Cavitation | 17 (21.0) | 11 (19.6) | 6 (24.0) | 0.66 |
| Consolidation | 14 (17.3) | 10 (17.9) | 4 (16.0) | 1 |
| Reticulointerstial pattern | 26 (32.1) | 16 (28.6) | 10 (40.0) | 0.31 |
| Miliary nodules | 0 | 0 | 0 | - |
| Lymphadenopathy | 14 (17.3) | 7 (12.5) | 7 (28.0) | 0.09 |
| Pleural effusion/empyema | 11 (13.6) | 6 (10.7) | 5 (20.0) | 0.30 |
| Any abnormality suggestive of active TB | 38 (46.9) | 23 (41.1) | 15 (60.0) | 0.12 |
| **Findings suggestive of prior TB** |  |  |  |  |
| Peri-bronchial fibrosis | 38 (46.9) | 21 (37.5) | 17 (68.0) | 0.011 |
| Nodular opacities | 27 (33.3) | 16 (28.6) | 11 (44.0) | 0.17 |
| Traction bronchiectasis | 29 (35.8) | 18 (32.1) | 11 (44.0) | 0.30 |
| Apical and upper lobe volume loss | 39 (48.2) | 23 (41.1) | 16 (64.0) | 0.06 |
| Calcified granulomas and/or lymph nodes | 11 (13.6) | 6 (10.7) | 5 (20.0) | 0.30 |
| Pleural thickening and/or calcification | 42 (51.9) | 29 (51.8) | 13 (52.0) | 0.99 |
| Any abnormality suggestive of prior TB | 58 (71.6) | 37 (66.1) | 21 (84.0) | 0.12 |
| Any abnormality suggestive of TB (active or prior) | 64 (79.0) | 42 (75.0) | 22 (88.0) | 0.18 |
